# Isolation and structural identification of a new T1-conotoxin with unique disulfide connectivities derived from *Conus bandanus*

**DOI:** 10.1590/1678-9199-JVATITD-2019-0095

**Published:** 2020-05-08

**Authors:** Nguyen Bao, Jean-Pière Lecaer, Ngo Dang Nghia, Phan Thi Khanh Vinh

**Affiliations:** 1 Faculty of Food Technology, Nha Trang University, 02 Nguyen Dinh Chieu, Nha Trang, Khanh Hoa, Vietnam.; 2 Institut de Chimie des Substances Naturelles, Centre de Recherche de Gif, FRC3115, UPR 2301, F-91198 Gif-sur-Yvette, France.; 3 Institute of Biotechnology and Environment, Nha Trang University, 02 Nguyen Dinh Chieu, Nha Trang, Khanh Hoa, Vietnam.

**Keywords:** T1-subfamily conotoxin, Conus bandanus, Bn5a, Disulfide connectivity, Cone snail venom

## Abstract

**Background:**

Conopeptides are neuropharmacological peptides derived from the venomous salivary glands of cone snails. Among 29 superfamilies based on conserved signal sequences, T-superfamily conotoxins, which belong to the smallest group, include four different frameworks that contain four cysteines denominated I, V, X and XVI. In this work, the primary structure and the cysteine connectivity of novel conotoxin of *Conus bandanus* were determined by tandem mass spectrometry using collision-induced dissociation.

**Methods:**

The venom glands of *C. bandanus* snails were dissected, pooled, and extracted with 0.1% trifluoroacetic acid in three steps and lyophilized. The venom was fractionated and purified in an HPLC system with an analytical reversed-phase C_18_ column. The primary peptide structure was analyzed by MALDI TOF MS/MS using collision-induced dissociation and confirmed by Edman's degradation. The peptide’s cysteine connectivity was determined by rapid partial reduction-alkylation technique.

**Results:**

The novel conotoxin, NGC_1_C_2_(I/L)VREC_3_C_4_, was firstly derived from *de novo* sequencing by MS/MS. The presence of isoleucine residues in this conotoxin was confirmed by the Edman degradation method. The conotoxin, denominated Bn5a, belongs to the T1-subfamily of conotoxins. However, the disulfide bonds (C_1_-C_4_/C_2_-C_3_) of Bn5a were not the same as found in other T1-subfamily conopeptides but shared common connectivities with T2-subfamily conotoxins. The T1-conotoxin of *C. bandanus* proved the complexity of the disulfide bond pattern of conopeptides. The homological analysis revealed that the novel conotoxin could serve as a valuable probe compound for the human-nervous-system norepinephrine transporter.

**Conclusion:**

We identified the first T1-conotoxin, denominated Bn5a, isolated from *C. bandanus* venom. However, Bn5a conotoxin exhibited unique C_1_-C_4_/C_2_-C_3_ disulfide connectivity, unlike other T1-conotoxins (C_1_-C_3_/C_2_-C_4_). The structural and homological analyses herein have evidenced novel conotoxin Bn5a that may require further investigation.

## Background

Conopeptides (conotoxins) are peptides derived from the venomous salivary glands of cone snails consisting of 8-84 amino acids and zero to five disulfide bridges. They are neuropharmacological probes and pharmacological development for G-protein-coupled receptors, ion channels (K^+^, Na^+^, Ca^2+)^, and neurotransmitter receptors (such as N-methyl-d-aspartate receptor, 5-hydroxytryptamine, nicotinic acetylcholine receptor) with high degrees of specificity and potency [1,2]. Recently, conopeptides were grouped into 29 superfamilies, based on conserved signal sequences with or without specific cysteine frameworks within each superfamily [3]. Among these, T-superfamily conotoxins that belong to the smallest group, typically 10-16 amino-acid residues in length, are widely distributed in all feeding types of *Conus* snails. These conotoxins include four different cysteine frameworks that contain four cysteines, namely "-CC-C-C-"(I), "-CC-CC-" (V), "-CC-C.[PO]C-" (X) and "-C-C-CC-" (XVI) [4].

So far, there are approximately 40 known sequences with cysteine framework V for all three species-based diet types, specifically piscivore, vermivore and molluscivore. Some T-conotoxins had been reported as pharmacological targets such as somatostatin receptors like CnVA [5], sodium channels like LtVd [6], presynaptic calcium channels like TxVA [7], and neuronal nicotinic acetylcholine receptors like TxVC [8]. All of them possess C_1_-C_3_/C_2_-C_4_ cysteine connectivities. Biological activities of conotoxins depend strictly on the peptide sequence and pairing of the cysteines. Matrix-assisted laser desorption/ionization time of flight (MALDI-TOF) mass spectrometry spectrometers combined with Edman degradation can provide the complete peptide sequence information from a small amount of sample. The fragmentation capabilities, such as collision-induced dissociation (CID) conferred by MALDI-TOF MS [9], along with the rapid partial reduction-alkylation procedure [10], are especially useful for determination of disulfide connectivity. In the present work, the reserved-phase chromatography was employed to enrich T-superfamily components from venom of mollusk-hunting cone snail species (*C. bandanus*). The sequence assignment of the peptide was determined using MALDI mass spectrometry. Furthermore, we established the unusable disulfide pairing of a novel T1-subfamily conotoxin using the partial reduction-alkylation procedure.

## Methods

### Isolation and purification of conopeptides

The specimens of each *C. bandanus* were collected from seawater at Ke Ga reef in Nha Trang Bay (Vietnam) and were frozen at -80 ºC. The venom of the whole *C. bandanus* apparatus was dissected, extracted with H_2_O/0.1% trifluoroacetic acid (TFA) in three steps, and lyophilized. The venom powder was dissolved and subjected to HPLC fractionation with a Shimadzu LC-class 10 HPLC system. The venom extract was purified by separation in an analytical reversed-phase C_18_ column (Vydac, 300Å, 5µm, 4.6 mm i.d.x250 mm) with solution A (0.1% TFA) and solution B (0.1% TFA in 90% CH_3_CN) as the mobile phase. The flow rate was maintained at 1 mL.min^-1^ with gradient program (0% of solution B for 10 min, then 0-50% of B for 45 min). The detection of peptides was monitored at the wavelength 220 nm. Further purification steps were carried out using gradients (8-13% of B in 7.5 min, then 13-18% of B in 169.5 min).

### Reduction and alkylation of disulfide bonds

Twenty µL of the purified fraction was reduced by incubation for 10 min at 65 °C in 40 µL of 20 mM tris (2-carboxyethyl) phosphine (TCEP) in 0.5 M 4-(2-hydroxyethyl)-1-piperazineethanesulfonic acid (HEPES). Alkylation was then achieved by the addition of 50 mM iodoacetamide (IAA) and incubated for 30 min at 25 °C in darkness. The mixture was lastly desalted by solid-phase extraction on a ZipTip C_18_ column (Millipore, Billerica, MA, USA).

### Rapid partial reduction and alkylation procedure

For rapid partial reduction [10], each 4 µL of Bn5a (2.15 mM) was reduced with 36 µL 20 mM TCEP in a 0.17 M sodium acetate buffer, pH ~3.0, for 2.5 min, immediately alkylated by 80 µL of 2.2 M IAA in 0.5 M Tri-acetate, 2 mM Na2-EDTA buffer (pH ~8.0) and incubated for 30 min in darkness. The whole reaction mixture was passed through an analytical C_18_ Vydac column to separate the different peptide forms. The eluents of different fractions were collected in Eppendorf tubes. The MALDI-TOF MS analysis was carried out to determine modified-peptide fraction(s) possessing only two carbamidomethyl cysteines. In the second reduction step, the modified peptide fractions were dried and incubated with 20 µL of 20 mM TCEP in 0.5 M HEPES (pH ~7.0) for 30 min. The finally modified peptide mixture was desalted with a Zip Tip C_18_ column.

### Mass spectrometry analysis

Mass spectrometry experiments were performed using a 4800 MALDI TOF/TOF™ Analyzer mass spectrometer (AB Sciex, Les Ulis, France). The samples were irradiated with an Nd:YAG laser operating at 355 nm wavelength, producing 3 ns wide pulses. The instrument was externally calibrated using a peptide mixture (peptide calibration 1 and 2 from ABSciex between 700 and 3700 Da). Acquisitions were performed in positive reflection mode. For the dried-droplet sample preparation method, 0.5 µL of the sample was mixed with 0.5 µL of a solution of 4 mg/ml of HCCA. For MS/MS experiments, precursor ions were accelerated at 8 keV, and the MS/MS spectra were acquired using 2 keV collision energy, with CID gas at a pressure of 3.5x10^-6^ Torr. Mass spectra were analyzed using Data Explorer 4.9 (AB Sciex). For peptide sequence analysis, the mass tolerance of the precursor was 10 ppm and 0.05 Da for fragment identification.

### Peptide sequencing by edman degradation

The amino-acid sequences of the native peptide were determined by automated Edman degradation using a Procise protein sequencer (Applied Biosystem model 492, Applied Biosystem, Foster City, CA, USA). Then 2 µL (~5.7 mM) of the native peptide was dissolved in 25 µL of 50% (v/v) aqueous TFA for sequencing.

## Results

### **Isolation of novel peptide from *C. bandanus* venom**

**Figure 1.**
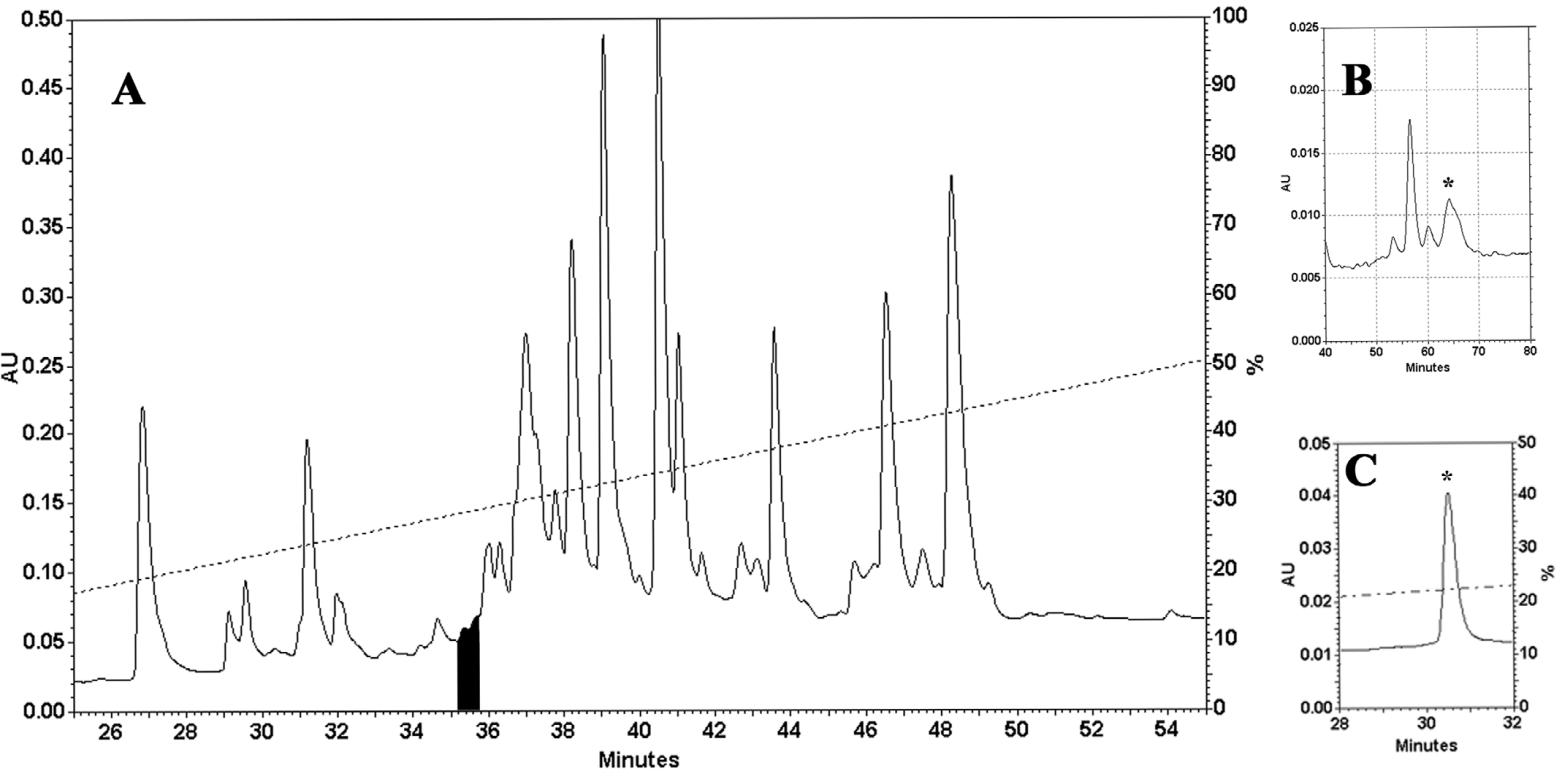
Isolation of novel peptide from *C. bandanus* venom: **(A)** RP-HPLC profile of *C. bandanus* venom. **(B)** Separation of the fraction highlighted in black. **(C)** Homogeneity inspection of the asterisk peak.

**Figure 2.**
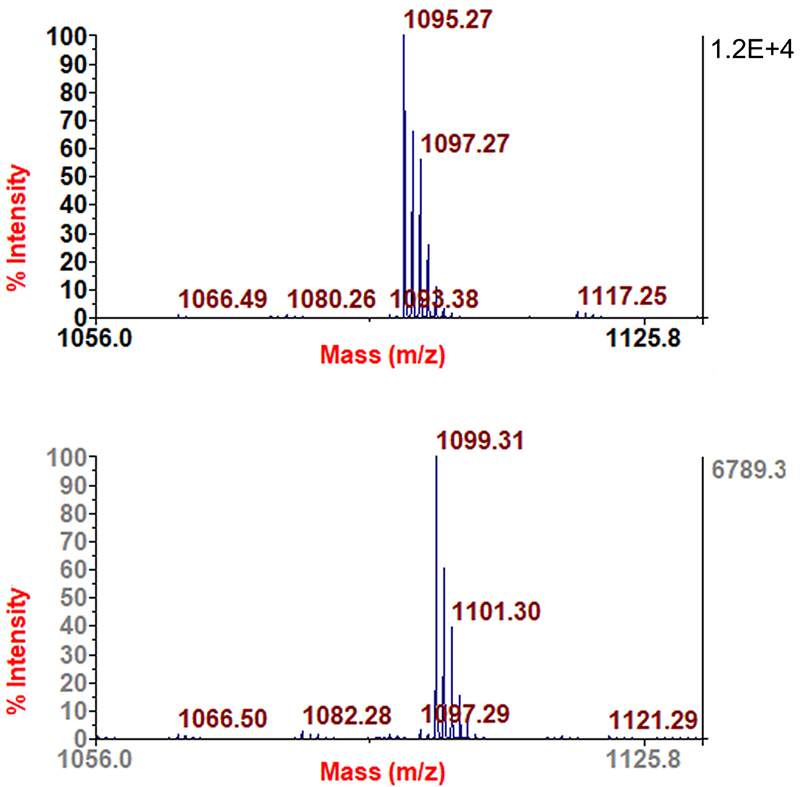
Determination of the cysteine number in the investigated native conopeptide p2.4.2 (upper graph) and in its reduced form by TCEP (lower graph) from off-line LC MALDI-TOF MS: Note the shift of 4 Da characterizing the reduction of two disulfide bonds.

In search of new conopeptides from the venom of *C. bandanus*, we have found a novel peptide containing an unusual arrangement of its disulfide connectivities. This peptide was collected from the throughput on an analytical C_18_ column between the 35^th^ and 36^th^ minutes (fraction highlighted in black, Figure 1A). The further separation of this fraction was carried out on the same column to collect the asterisk peak (Figure 1B). This step was repeated until the end of the sampling. This asterisk peak was utilized for testing the proximate homogeneity of the peptide (Figure 1C). MALDI-TOF MS analysis was used for peptide investigation, which showed a [M+H]^+^ species, detected at *m/z* 1095.27, that characterized a toxin with molecular mass of 1094.26 Da. Following total reduction with TCEP, the [M+H]^+^ species was detected at *m/z* 1099.31, which indicated the presence of two disulfide bonds in Bn5a (net increase of 1Da for each cysteine involved in a disulfide bridge, Figure 2).

### Primary structure determination

**Figure 3.**
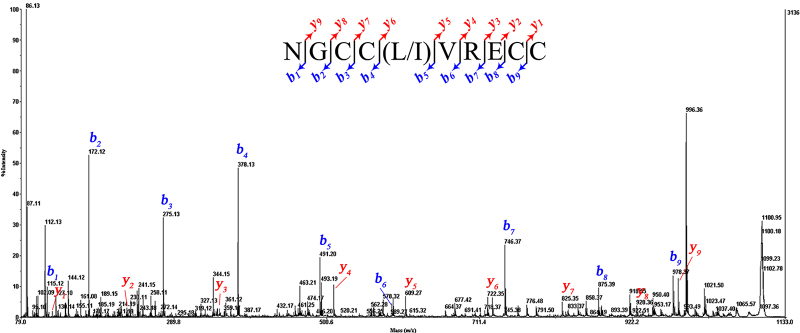
Primary structure determination of CID mass spectrum profile of reduced-Bn5a form, recorded with the MALDI-TOF/TOF 4800 mass spectrometer: The inset shows the sequence derived from these MS/MS spectra. Note *m/z* 86.13 corresponds to the immonium ion of Leu or Ile.

The primary structure was preliminarily investigated by the MS/MS technique, using CID fragmentation that generated predominantly *b-* and *y-*type product ions. Figure 3 showed the CID mass spectra of completely reduced peptide. This spectrum of *m/z* 1099.31 parent ions revealed its fragmentation in the series of *b-* and *y-*type ions, from position 1 to position 9. From the differences between the most intense product ions of *b*-type series (*b*_*1*_ to *b*_*7*_ ), the initial tag of the sequence was characterized as GCC(L/I)VR. There was an ambiguity in distinguishing between Leu and Ile residues (mass of 113 Da) at position 5. Following this initial tag and after the Glu (E), and Cys (C) residues were determined, respectively, through differences between the product ions *b*_*7*_ /*b*_*8*_ and *b*_*8*_ /*b*_*9*_ . Based on the mass analysis and the combination of the theoretical monoisotopic mass, we inferred an Asn residue at the N-terminus and a Cys residue (not amidated) at the C-terminus of the peptide. The product ions of the *y*-type series (*y*_*1*_ to *y*_*9*_ ) were all observed with lower signal intensity than *b*-type series. Thus, the initial sequence assignment of *m/z* 1099.31 parent ion was NGCC(L/I)VRECC exhibiting a cysteine framework V (-C_1_C_2_-C_3_C_4_-) of the T-superfamily of conopeptides, which is denominated Bn5a according to the nomenclature of conotoxins [11]. The monoisotopic molecular mass of Bn5a (1094,26) matched well with the calculated theoretical data (1094,37).

**Figure 4.**
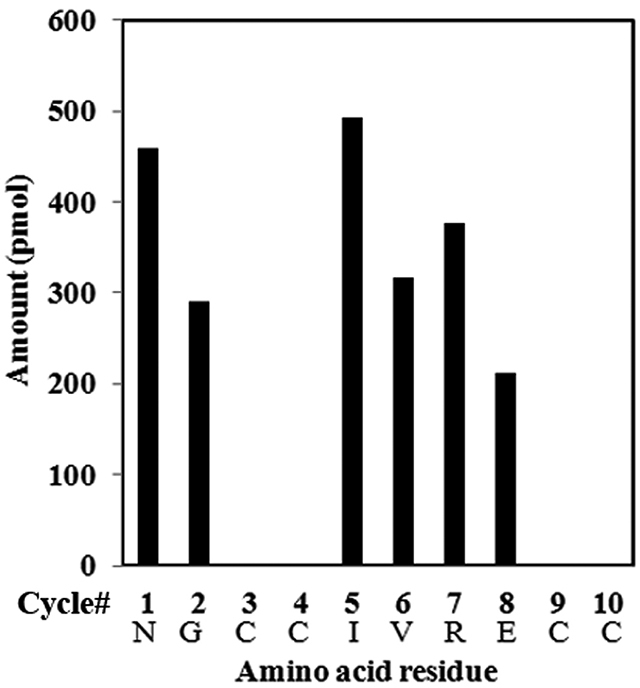
Solid-phase Edman degradation of native Bn5a.

Automated Edman sequencing of the native peptide confirmed the peptide-sequencing result and yielded an unambiguous 10-residue sequence (Figure 4) with 4-phenylthiohydantoin (PTH)-cysteine residues at positions 3, 4, 9 and 10, which were not recorded in this method but were identified by MALDI TOF/TOF CID MS/MS. At position 5, we could confirm the isoleucine residue having an amount of ~500 pmol in place of leucine. Thus, sequence Bn5a has a total of 10 amino acids with two disulfide bridges and a free C-terminal Cys residue. The complete linear Bn5a sequence is NGCCIVRECC.

### Disulfide connectivities

The native Bn5a conotoxin was partially reduced and immediately followed by alkylation with IAA in 0.5 M Tri-acetate, 2 mM Na2-EDTA buffer (see in the methods section). Through this approach, alkylation was affected by IAA resulting in a mass increase of 58 Da per sulfhydryl group. The differentially labeled peptide fractions were separated and collected on a C_18_ Vydac analytical column (see Additional file 1). We obtained seven peaks with different retention times on the analytical C_18_ column. Among them were three partially labeled peaks (at 35, 38.5 and 39 minutes), in which one cysteine bridge remains intact ([M+H]^+^ species detected at *m/z* 1211.1). However, the 39^th^ min fraction was so small that we were unable to characterize its structure further. Two remaining fractions were then entirely reduced by TCEP. After that, they were desalted on a C_18_ ZipTip column and then subjected to MS analysis and CID MS/MS fragmentation. 

**Figure 5.**
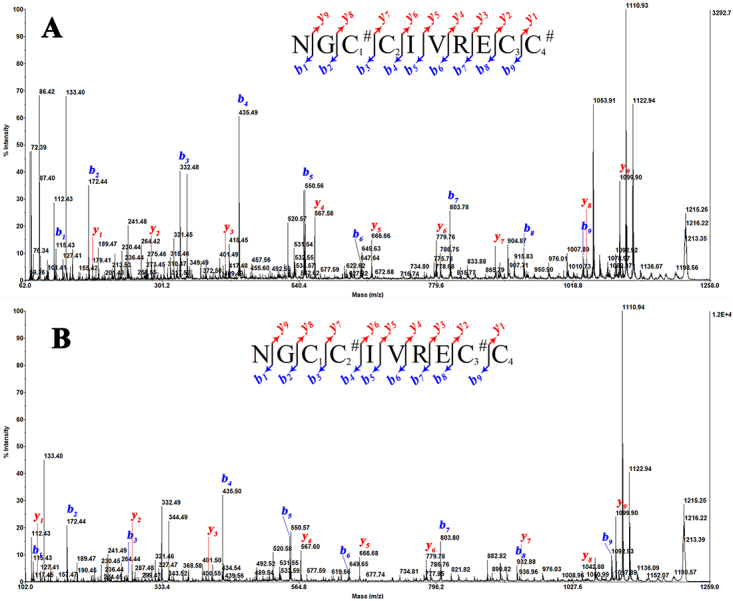
Determination of Bn5a-cysteine connectivity: **(A)** CID mass spectrum of C_1_-C_4_ IAA-labeled Bn5a and (B) C_2_-C_3_ IAA-labeled Bn5a from experiments partially reduced by alkylation. Note #: alkylated cysteine by IAA.

**Figure 6.**
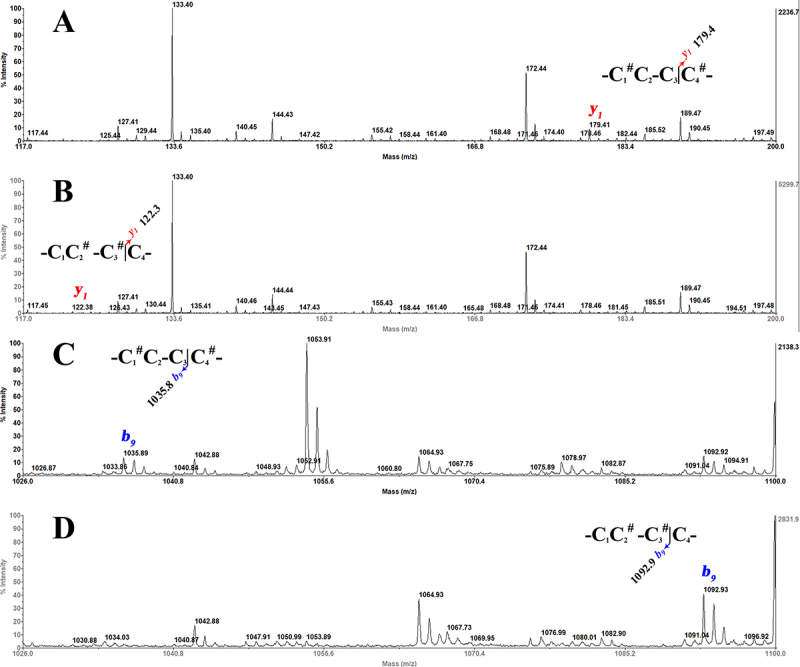
Close-up of the key fragment ions between two modified species in the determination of Bn5a cysteine-connectivity: A portion of the CID mass spectrum of C_1_-C_4_ IAA-labeled Bn5a **(A, C)** and C_2_-C_3_ IAA-labeled Bn5a **(B, D)** from experiments partially reduced by alkylation. **(A, B)** edited between *m/z* 117 and 200, showing *y*_*1*_ -ions; **(C, D)** edited between *m/z* 1026 and 1100, showing *b*_*9*_ -ions; Note #: alkylated cysteine by IAA.

Figure 5 shows the CID MS/MS spectra of the two possible rapidly alkylated species ([M+H]^+^ = 1213.45 Da), which predominantly generated *b-* and *y-*type ions. Figure 5A showed that the species contained C_1_ and C_4,_ which were modified with IAA through *b*_*2*_ */y*_*8*_ -*b*_*3*_ */y*_*7*_ and *b*_*9*_ */y*_*1*_ ions. The other species possessed C_2_ and C_3,_ which were modified with IAA (Figure 5B) through *b*_*3*_ */y*_*7*_ *-b*_*4*_ */y*_*6*_ and *b*_*8*_ */y*_*2*_ *-b*_*9*_ */y*_*1*_ ions. It is worth noting that performing rapid partial reduction-alkylation procedures generated not only completely alkylated species but also species having three alkylated-cysteine scramblings that could be separated on the C_18_ analytical column (see Additional file 2). Furthermore, two differently modified species shared almost total commonality of fragments, but some essential fragment ions, such as *b*_*9*_ -/*y*_*1*_ -ions, were distinguishable and are displayed clearly in Figure 6.

**Figure 7.**
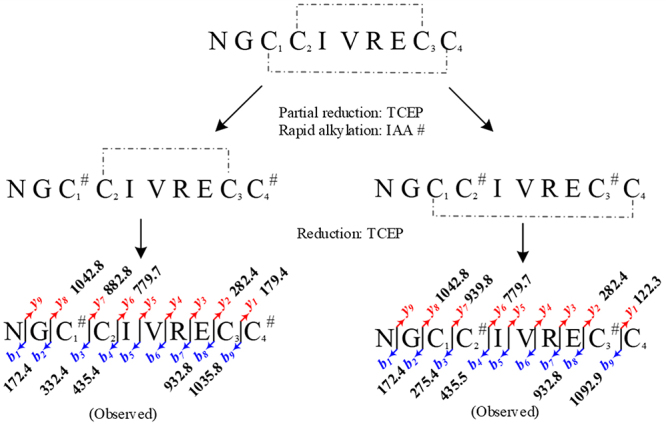
Overview of Bn5a-cysteine connectivity determination and highlight of the key fragments.

Figure 7 provides an overview of this approach adopted and shows the essential fragments, which permit the unambiguous determination of the cysteine connectivities. Both possible rapidly alkylated species with the same *m/z* 1213.45 were identifiable. These data confirm that the disulfide bonds in the Bn5a conopeptide are C_1_-C_4_ and C_2_-C_3._

## Discussion

A homology comparison reveals that the sequence of Bn5a belongs to the T-superfamily, more specifically, T1-subfamily conotoxins (Table 1). This class of conotoxins has a common feature of the presence of CC-motif at the N- or C-termini of the molecule. These conotoxins in Table 1 possess four residues presented between the CC pair, isolated from all three species-based diet types, in which the majority of snails are molluscivorous or vermivorous. So far, 19 known sequences possess the -CC-x(4)-CC- motif, while three conotoxins (Bn5a, MrVA, Mo1274) share a common feature, namely the presence of a CC pair at the C-terminus of the molecule. Remarkably, Mo1274 is a bromotryptophan-containing conopeptide, isolated from the venom of a vermivorous *Conus monile* [12]. This Bn5a peptide is the first T1-conotoxin isolated from *C. bandanus* venom. There is a notable exception for Bn5a and MrVA (from another mollusk-hunting *C. marmoreus* venom), which share 80% apparent homology on the protein sequence level. The reason for high consensus could be relatively close species from both conchological and phylogenetic perspectives between *C. bandanus* and *C.marmoreus* [13,14]*.*

**Table 1.** T1-subfamily conopeptides, isolated from different *Conus* species

	Name	Organism (diet)	Mature sequence	Reference
1	Bn5a	*C. bandanus* (m)^b^	NG**CC** I VRE**CC**	This work
2	MrVA	*C. marmoreus* (m)	NA**CC** I VRQ**CC**	[15]
3	Mr5.6	*C. marmoreus* (m)	NG**CC**RAGD**CC**S	[16]
4	Qc5.1	*C. quercinus* (v)	G**CC** ARLT**CC**V	[17]
5	Pu5.2	*C. pulicarius* (v)	G**CC**EDKT **CC**FI*	[18]
6	Ca5.4	*C. caracteristicus* (v)	**CC**PNKP **CC**FI	[17]
7	VcVA	*C. victoriae* (m)	CCPGKOCCRI*	[19]
8	G5.4	*C. geographus* (p)	D**CC**EERW**CC**F	[20]
9	Ts-011	*C. tessulatus* (v)	G**CC**EDKT**CC**FI*	[21]
10	Qc5.2	*C. quercinus* (v)	G**CC**AMLT**CC**V	[17]
11	TxMRCL-03	*C. textile* (m)	N**CC**RRQ I**CC**GRPS	[21]
12	Vc5.7	*C. victoriae* (m)	E**CC**EDGW**CC**TAAPLTAP	[22]
13	LeDr192	*C. litteratus* (v)	E**CC**EDGW**CC**TAAPLT*	[23]
14	TxVA	*C. textile* (m)^a^	E **CC** EDGW **CC** TAAO	[24]
15	Pu5.3	*C. pulicarius* (v)	S**CC**P E EP**CC**FW	[18]
16	Pn-B02	*C. pennaceus* (m)	E**CC**SDGW**CC**PA*	[21]
17	TeAr193	*C. textile* (m)	N**CC**RRQ I**CC**GRT	[23]
18	Vc5.9	*C. victoriae* (m)	RN**CC**RLQ I **CC**GRT	[22]
19	Mo1274	*C. monile* (v)^a^	GNW **CC**SARV CC*	[12]

W: bromotryptophan; T: glycosylated threonine; O: 4-Hydroxyproline; E: gamma carboxylic glutamic acid; *: amidated C-terminus; (a): disulfide connectivity: C_1_-C_3_/C_2_-C_4_; (b): disulfide connectivity: C_1_-C_4_/C_2_-C_3_. (m): molluscivorous type; (v): vermivorous type; (p): piscivorous type.

So far, the T-superfamily conotoxins found in the venom ducts of all three feeding types of *Conus* include four cysteine frameworks, specifically "C_1_C_2_-C_3_-C_4_"(I), "C_1_C_2_-C_3_C_4_" (V), "C_1_C_2_-C_3_-C_4_" (X) and "C_1_-C_2_-C_3_C_4_" (XVI) [4,5]. Among them, T1-conotoxins with framework V possess 1-3, 2-4 cysteine connectivities, while both framework-X and framework-I conotoxins present 1-4 and 2-3 cysteine pairings. However, there were no data on disulfide connectivities for frameworks XVI. The Bn5a possesses four Cys residues and two disulfide bridges resulting in three possible disulfide pairing patterns, namely C_1_-C_3_/C_2_-C_4_, C_1_-C_4_/C_2_-C_3_ and C_1_-C_2_/C_3_-C_4_. It may be noted that the C_1_-C_2_/C_3_-C_4_ arrangement, which requires disulfide formation between contiguous Cys residues, is relatively rare. 

Echterbille et al. [25] observed the partial reduction of conopeptides having two disulfide bridges (using TCEP 400 µM for 30 min at pH 4.5 or 2) to assign disulfide bridge arrangements. This approach could lead to the scrambling of disulfides in the observed peptides. Herein, we used a rapid partial-reduction/alkylation procedure to characterize the native fold of Bn5a. It is said that our method is the same as that of Echterbille et al. [25], but in contrast we applied partial reduction in a shorter time (2.5 min vs. 30 min). We performed the partial reduction at pH 3 for 2.5 min and immediately alkylated by saturated IAA in the buffer solution (pH ~8.0) to maximally prevent interchange and/or reoxidation. The disulfide scrambling phenomenon, in our opinion, is impossible in the alkylation step. A small number of species may present disulfide scrambling in the rapid partial reduction procedure. Thus, we observed this scrambling at the 39-min peak (see Additional file 1).

Fortunately, we successfully collected two isomers. Each of isomers contained one disulfide bond and two alkylated cysteines at the 35- and 38.5-min peaks. The disulfide connectivities of the 35-min peak had been proven with the pattern C^#^C-CC^#^ (Figure 5A) while the connectivities of the 38.5-min peak presented the CC^#^-C^#^C pattern (Figure 5B). From these results, the reaction time of rapid partial reduction should be reduced to 1-1.5 min. It could help to decrease the number of reduced species and disulfide-scrambling species. Furthermore, the alkaline condition of the alkylation step should also be adjusted to the acidic condition (pH 2-3) to prevent the reduction of remaining disulfide bond(s) of peptides.

**Figure 8.**
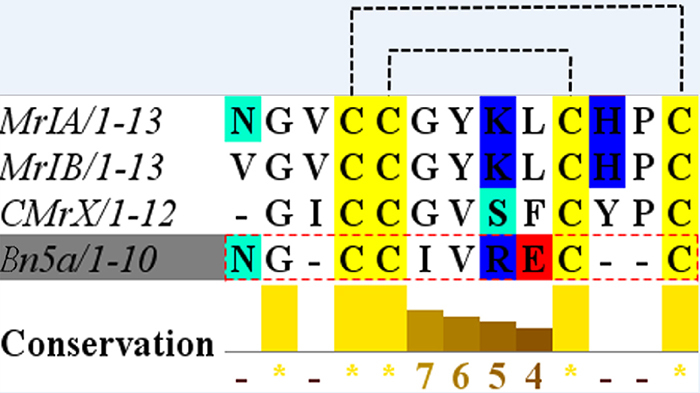
Multiple sequence alignment of Bn5a and three other bioactive compounds with different cysteine frameworks: Residue conservation score was calculated via the software Jalview v2.8. The dotted line indicates disulfide connectivity. Gaps have been presented to optimize the alignment sequence identity. Color coding employs the following scheme: hydrophobic residues are in white, negatively charged residues in red, positively charged residues in blue, polar uncharged residues in green, and cysteine residues in yellow. The color intensity and the “conservation index” score (1-11) reflect the conservation of physicochemical properties of amino acids in the particular column of the alignment. *conserved column (where the highest score is 11).

Of the 19 listed sequences of T1-conotoxins possessing the -CC-x(4)-CC- motif, the cysteine connectivity has been established only in the case of three peptides, including the peptide investigated in the present work. The Bn5a belonged to framework V (C_1_C_2_-C_3_C_4_). However, it possessed only C_1_-C_4_/C_2_-C_3_ cysteine connectivities unlike the reported conopeptides sharing the same framework V (1-3, 2-4 cysteine connectivities), such as Mo1274 and TxVA. Additionally, it shared the same disulfide pairing of conopeptides of the frameworks I and X, namely MrIA, MrIB [26] and CMrX [27], respectively (Figure 8), while chi-MrIA is a 13-residue peptide in the *C. marmoreus* venom that had been found to act as antidepressant inhibitors of the norepinephrine transporter (NET) in both mice and humans [28]. 

CMrX conotoxin caused breathing difficulty, flaccid paralysis, and death in 2 hours at the dose 12.6 µg/g of body weight. Figure 8 displays the alignment analysis with high homology, hydrophobicity, charged distribution of Bn5a conotoxin and three other bioactive conotoxins [29]. The sequences Bn5a and MrIA share the most common physicochemical properties that could induce the same cysteine connectivities. It is suggested that Bn5a may target the NET. This transporter is widely expressed in the human nervous system and plays an essential role in regulating norepinephrine signaling and homeostasis by transporting synaptically released norepinephrine back into the presynaptic neuron [30]. Dysregulation of the removal of norepinephrine by NET is associated with many neuropsychiatric diseases such as depression, anxiety disorders, attention deficit hyperactivity disorder, and epilepsy [31]. Further investigation of the biological activity of this unique conotoxin may reveal its pharmacological properties.

## Conclusion

In summary, the purification and mass spectral characterization of a novel peptide, Bn5a, isolated from the venom of a molluscivorous snail, *C. bandanus*, were described. The primary structure NGCCIVRECC of the peptide was determined through *de novo* sequencing by tandem mass spectrometry and subsequently by Edman degradation. Based on a determination of the cysteine framework, intervening residues, and homology comparison, Bn5a was classified in the T1-subfamily of conotoxins. This peptide was the first T1-conotoxin isolated from the *C. bandanus* venom. Moreover, Bn5a possessed -C_1_C_2_-x(4)-C_3_C_4_- pattern belonging on the framework V but exhibited special C_1_-C_4_/C_2_-C_3_ disulfide connectivities that differed from the disulfide connectivity patterns in other T1-conotoxins of the framework V (C_1_-C_3_/ C_2_-C_4_). The difference in structure may suggest a specific property in pharmaceutical function.

### Abbreviations

 CID: collision-induced dissociation; HCCA: cyano-4-hydroxycinnamic acid; HEPES: 4-(2-hydroxyethyl)-1-piperazineethanesulfonic acid; HPLC: reversed-phase high-performance liquid chromatography; IAA: iodoacetamide; MALDI: matrix-assisted laser desorption/ionization; MS/MS: tandem mass spectrometry; MS: mass spectrometry; Na2-EDTA: ethylenediaminetetraacetic acid disodium salt dihydrate; NET: norepinephrine transporter; PTH: phenylthiohydantoin; TCEP: tris (2-carboxyethyl) phosphine; TFA: Trifluoroacetic acid; TOF: time of flight.

## Supplementary material

The following online material is available for this article:

Additional file 1. RP-HPLC profile of rapid partial reduction-alkylation procedure of Bn5a peptide. Note: [M+H]^+^ of Bn5a with four alkylations: 1327.21 Da; [M+H]^+^ of Bn5a with three alkylations: 1270.2 Da; [M+H]^+^ of Bn5a with two alkylations and one bridge: 1211.1 Da; [M+H]^+^ of native Bn5a: 1095.14 Da.

Additional file 2. Spectra of the Bn5a species having three alkylated-cysteines at 32^nd^ min (upper graph) and 34^th^ min (lower graph).
