# Supplementary material for: Isolation and structural identification of a new T1-conotoxin with unique disulfide connectivities derived from Conus bandanus
Source: J Venom Anim Toxins Incl Trop Dis. 2020 May 8;26:e20190095. doi: 10.1590/1678-9199-JVATITD-2019-0095 (PMC7216822; doi:10.1590/1678-9199-JVATITD-2019-0095)
Supplement: Additional file 1. [file 1678-9199-jvatitd-26-e20190095-s1.pdf]

Supplementary material to “Isolation and structural identification of a new T1-conotoxin with unique disulfide connectivities derived from *Conus bandanus”*

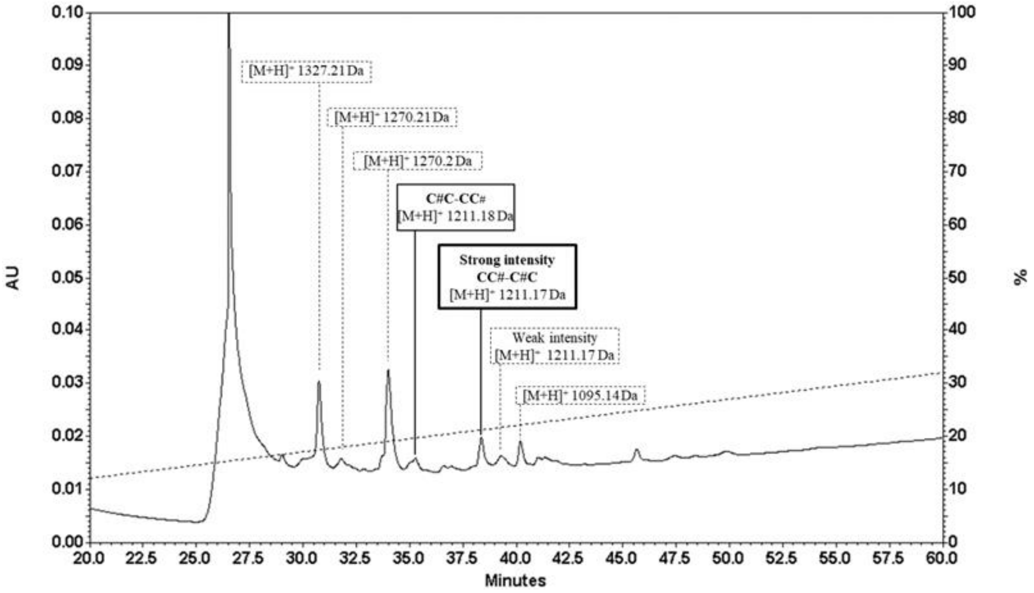

Additional file 1. RP-HPLC profile of rapid partial reduction-alkylation procedure of Bn5a peptide. Note: $[M+H]^+$ of Bn5a with four alkylations: 1327.21 Da; $[M+H]^+$ of Bn5a with three alkylations: 1270.2 Da; $[M+H]^+$ of Bn5a with two alkylations and one bridge: 1211.1 Da; $[M+H]^+$ of native Bn5a: 1095.14 Da.
