# Supplementary material for: Isolation and structural identification of a new T1-conotoxin with unique disulfide connectivities derived from Conus bandanus
Source: J Venom Anim Toxins Incl Trop Dis. 2020 May 8;26:e20190095. doi: 10.1590/1678-9199-JVATITD-2019-0095 (PMC7216822; doi:10.1590/1678-9199-JVATITD-2019-0095)
Supplement: Additional file 2. [file 1678-9199-jvatitd-26-e20190095-s2.pdf]

Supplementary material to "Isolation and structural identification of a new T1-conotoxin with unique disulfide connectivities derived from *Conus bandanus*"

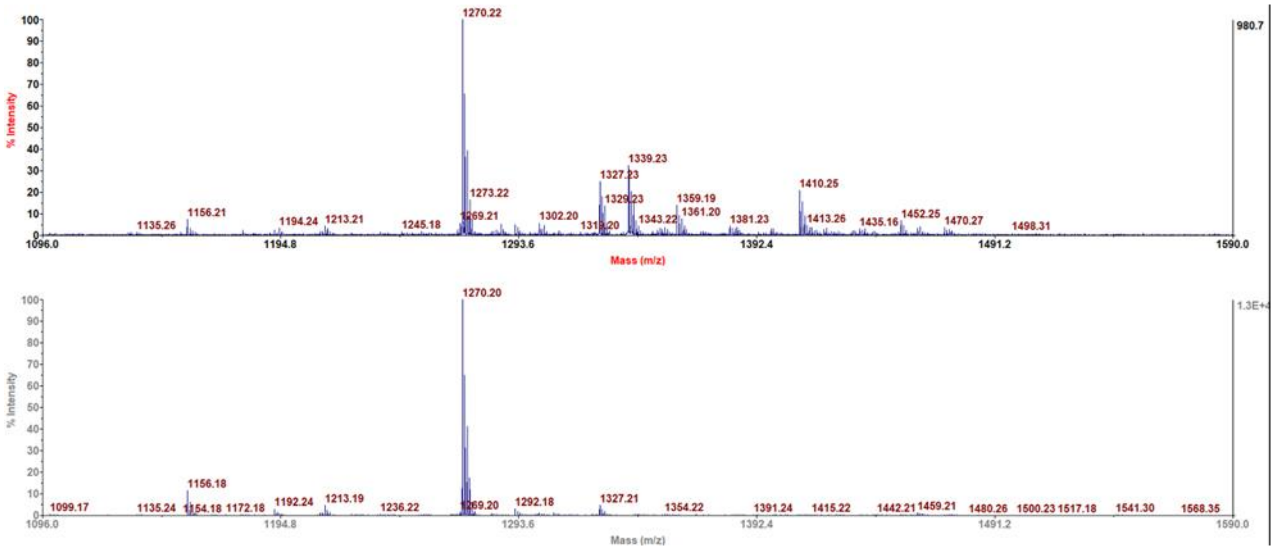

Additional file 2. Spectra of the Bn5a species having three alkylated-cysteines at 32nd min (upper graph) and 34th min (lower graph).
